# Nutritional Recovery with a Soybean Diet after Weaning Reduces Lipogenesis but Induces Inflammation in the Liver in Adult Rats Exposed to Protein Restriction during Intrauterine Life and Lactation

**DOI:** 10.1155/2015/781703

**Published:** 2015-03-29

**Authors:** Sílvia Regina de Lima Reis, Naoel Hassan Feres, Leticia Martins Ignacio-Souza, Roberto Vilela Veloso, Vanessa Cristina Arantes, Nair Honda Kawashita, Edson Moleta Colodel, Bárbara Laet Botosso, Marise Auxiliadora de Barros Reis, Márcia Queiroz Latorraca

**Affiliations:** ^1^Departamento de Alimentos e Nutrição, Faculdade de Nutrição, Universidade Federal de Mato Grosso (UFMT), 78060-900 Cuiabá, MT, Brazil; ^2^Departamento de Química, Instituto de Ciências Exatas e da Terra, Universidade Federal de Mato Grosso (UFMT), 78060-900 Cuiabá, MT, Brazil; ^3^Departamento de Clínica Médica Veterinária, Faculdade de Agronomia e Medicina Veterinária, Universidade Federal de Mato Grosso (UFMT), 78060-900 Cuiabá, MT, Brazil

## Abstract

We evaluated the effects of postweaning nutritional recovery with a soybean flour diet *on de novo* hepatic lipogenesis and inflammation in adult rats exposed to protein restriction during intrauterine life and lactation. Rats from mothers fed with protein (casein) in a percentage of 17% (control, C) or 6% (low, L) during pregnancy and lactation were fed with diet that contained 17% casein (CC and LC groups, resp.) or soybean (CS and LS groups, resp.) after weaning until 90 days of age. LS and CS rats had low body weight, normal basal serum triglyceride levels, increased ALT concentrations, and high HOMA-IR indices compared with LC and CC rats. The soybean diet reduced PPAR*γ* as well as malic enzyme and citrate lyase contents and activities. The lipogenesis rate and liver fat content were lower in LS and CS rats relative to LC and CC rats. TNF*α* mRNA and protein levels were higher in LS and CS rats than in LC and CC rats. NF-*κ*B mRNA levels were lower in the LC and LS groups compared with the CC and LC groups. Thus, the soybean diet prevented hepatic steatosis at least in part through reduced lipogenesis but resulted in TNF*α*-mediated inflammation.

## 1. Introduction

The liver has an important role in lipid metabolism, which includes the mobilization and synthesis of free fatty acids as well as the storage and export of lipids and lipoproteins [1]. Various drugs, nutritional factors, and genetic defects in energy metabolism can result in excess hepatic triacylglycerol accumulation (hepatic steatosis) [2]. Hepatic steatosis can be a benign condition, or it may evolve with inflammation (steatohepatitis), fibrosis, and cirrhosis, an altered spectrum termed nonalcoholic fatty liver disease (NAFLD) [3]. The most common disorder associated with NAFLD is insulin resistance [4].

Insulin resistance is a condition wherein higher than normal insulin levels are needed to provoke normal metabolic responses or where normal metabolic responses are not achieved with normal insulin concentrations. Depending on the primary site of involvement, the insulin resistance can be central (liver) or peripheral (muscle or fat tissue) [5]. In NAFLD, the initial site appears to be in the periphery, followed by or resulting in hepatic steatosis, which exacerbates hepatic insulin resistance and thus the degree of overall insulin resistance [6].

Peripheral insulin resistance increases the serum levels of free fatty acids derived from the lipolysis of triacylglycerol from white adipose tissue [7]; these fatty acids are taken up by the liver and used in the production of triacylglycerol [8]. In addition, chronic hyperinsulinemia resulting from overall insulin resistance promotes* de novo* hepatic lipogenesis through the upregulation of lipogenic transcription factors [2]. A transcription factor that participates in the development of fatty liver in rodents is SREBP-1c (sterol regulatory element-binding protein-1c) [9]. In the nucleus, SREBP-1c transcriptionally activates genes required for lipogenesis; this effect is mediated by insulin [10]. Peroxisome proliferator-activated receptor gamma (PPAR*γ*) is another transcription factor involved in the development of hepatic steatosis in rodents [2]. In the liver, PPAR*γ* is usually expressed at very low levels [11], but its expression is markedly increased in animal models exhibiting insulin resistance and fatty livers [12].

Increases in oxidative stress and in factors that promote proinflammatory cytokine expression, such as interleukin (IL)-6 [13] and tumor necrosis factor-*α* (TNF*α*) [14], are implicated in the development of nonalcoholic steatohepatitis (NASH), with the nuclear factor kappa B (NF-*κ*B) playing a critical role in the modulation of proinflammatory transcription [15]. Moreover, several studies have documented the association of insulin resistance (liver and adipose tissue) with inflammation [16].

Protein restriction is associated with hepatic steatosis [17]. In our laboratory, we have studied an experimental model of protein restriction in early life that demonstrates deficits in insulin secretion [18] and liver insulin resistance [19] in adulthood. We use soybean flour diets as an alternative low-cost and high-quality protein source to feed these animals in an attempt to prevent and/or to treat the long-term consequences of malnutrition [20]. This choice was motivated by scientific evidence showing that the consumption of soy protein and isoflavones may exert beneficial effects on glucoregulation, lipotoxicity in the liver, and lipidemia by acting on a wide spectrum of biochemical and molecular activities [21, 22].

Interestingly, we observed that serum insulin concentrations were increased in animals reared on a soybean diet, but alterations occurred in the early steps of the hepatic insulin signal transduction pathway, indicating hepatic insulin resistance [23, 24]. Moreover, the weight and lipid content of the white adipose tissue as well as the lipolysis rate by isoproterenol in white adipocytes were decreased in rats fed the soybean diet [25]. Thus, the aim of this study was to investigate the effects of nutritional recovery after weaning with a soybean flour diet on* de novo* hepatic lipogenesis and inflammation in adult rats exposed to protein restriction during intrauterine life and lactation.

## 2. Materials and Methods

### 2.1. Animals and Diets

The experimental procedures involving rats were performed in accordance with the guidelines of the Brazilian College for Animal Experimentation (COBEA) and were approved by the ethics committee at the Federal University of Mato Grosso. Male and virgin female Wistar rats (85–90 days old) were obtained from the university's own breeding colony. Mating was performed by housing males with females overnight (1 male and 4 females), and pregnancy was confirmed by the presence of sperm in vaginal smears. Pregnant females were separated at random and maintained from the first day of pregnancy until the end of lactation on isocaloric diets containing 6% (low protein (LP) diet, *n* = 6) or 17% (control (C) diet, *n* = 8) protein. Spontaneous delivery occurred at day 22 of pregnancy, after which, at 3 days of age, large litters were reduced to eight pups to ensure a standard litter size per mother. After weaning in the 4th week, the males were divided into five groups: CC, consisting of offspring born to and suckled by mothers fed a C diet and subsequently fed the same diet after weaning until 90  days of age; CS, consisting of offspring born to and suckled by mothers fed a C diet and subsequently fed a soybean flour diet with 17% protein after weaning until 90  days of age; LL, consisting of offspring born to mothers fed an LP diet and subsequently fed the same diet after weaning until 90  days of age; LC, consisting of offspring born to mothers fed an LP diet and subsequently fed a C diet after weaning until 90  days of age; and LS, consisting of offspring born to mothers fed an LP diet and subsequently fed a soybean flour diet containing 17% protein after weaning until 90  days of age. The diets have been previously described [23, 26]. Throughout the experimental period, the rats were given free access to food and water. The rats were weighed at birth and at 30 and 90  days. All experimental procedures were performed at 90  days of age.

### 2.2. Sample Collection and Analyses

The homeostasis model assessment of insulin resistance (HOMA-IR) index was assessed from the basal glucose and insulin concentrations using the following formula: basal glucose (mmol/L) × basal insulin (*μ*U/mL)/22.5.

Fed rats were euthanized, and liver tissue samples were quickly removed, frozen immediately in liquid nitrogen, and stored at –80°C to determine malic enzyme (ME) and citrate lyase (CLY) activities; hepatic fat content; and nSREBP-1c (nuclear sterol regulatory element-binding protein-1c), PPAR*γ*, ME, CLY, TNF*α*, NF-*κ*B, and IL-10 expression levels by immunoblotting. The mRNA expression levels of TNF*α*, IL-6, IL-10, and NF-*κ*B were determined by real-time PCR. The remainder of the liver was removed and preserved for histologic analysis. Liver samples were obtained for* in vivo* lipogenesis measurements from another group of rats in the fed state. Biochemical and hormonal measurements were obtained in the fasting state. Blood glucose concentrations were determined using a portable glucose meter (Accu-Chek, Roche Diagnostics, Mannheim, Germany). After euthanasia, aliquots of serum obtained by centrifugation were used to measure total serum protein [27], serum triacylglycerol, and hepatic aminotransferase concentrations (BT-3000 Plus, Wiener Lab, Rosario, Argentina). Serum insulin concentrations were determined by radioimmunoassay [28].

### 2.3. Measurement of Enzymatic Activity in the Liver

ME activity was assayed by the method of Ochoa [29] in accordance with the modifications proposed by Hsu and Lardy [30]. CLY activity was assayed as described by Srere [31].

### 2.4. Liver* In Vivo* Fatty Acid Synthesis

^3^H_2_O (3 mCi) dissolved in 0.3 mL saline was administered to anesthetized rats by intraperitoneal injection 1 h before the experiment. The liver tissue was immediately removed and weighed. Total lipid from the liver was evaluated according to Folch et al. [32]. ^3^H_2_O was removed from the inferior phase (predominantly chloroform) by washing three times with a superior-phase mixture. After each shaking, the tubes were briefly centrifuged to sharpen the phase boundary, and the superior phase was aspirated and discarded. Isolation and counting of ^3^H_2_O-labeled fatty acids from the inferior phase was performed as previously described [33]. For the calculations of lipid synthesis in experiments with ^3^H_2_O, it was assumed that the specific activity of intracellular water was identical to that of plasma water, which was determined directly in aliquots of diluted (20 times) plasma. The rates of tissue lipid synthesis were calculated assuming that each fatty acid incorporated into triacylglycerol contained 13.3 atoms of tritium.

### 2.5. Liver Histology

Paraffin-embedded sections were stained with hematoxylin and eosin. Histologic evaluation included a semiquantitative analysis of the presence of micro- and macrovesicular fat. All sections were coded and analyzed blindly by the pathologist without knowledge of related characteristics or diet. The degree of fat accumulation was graded on a scale of 0 to 4 as follows: 0 = no evidence of or barely visible microvesicular fat; 1+ = <25%; 2+ = 25% to 49%; 3+ = 50% to 75%; 4+ = fat involving >75% of the lobule.

### 2.6. Immunoblot Analysis of ME and CLY in Total Liver Extracts

One frozen liver fragment was homogenized in freshly prepared buffer (1% Triton X-100, 100 mmol/L Tris-HCl (pH 7.4), 100 mmol/L sodium pyrophosphate, 100 mmol/L sodium fluoride, 10 mmol/L EDTA, 10 mmol/L sodium orthovanadate, 2.0 mmol/L PMSF, and 0.1 mg aprotinin/mL). Insoluble material was removed by centrifugation for 20 min at 12000 g at 4°C. Protein concentrations were determined by the Bradford method [34]. Samples containing 40 *μ*g of protein from each experimental group were incubated with 4x concentrated Laemmli sample buffer [35] and 15 mg of DTT and assayed on polyacrylamide gels at 120 V for 90 min (10% gels for ME and CLY). The electrotransfer of proteins to nitrocellulose membranes (Bio-Rad) was per formed for 90 min at 120 V in buffer containing methanol and SDS. After ascertaining transfer efficiency by Ponceau staining, the membranes were blocked with 5% albumin in Tween-Tris buffered saline (TTBS) (10 mmol/L Tris, 150 mmol/L NaCl, 0.5% Tween 20) overnight at 4°C. ME, CLY, TNF*α*, NF-*κ*B, and IL-10 were detected in the membranes after a 2 h incubation at room temperature with anti-ME1 rabbit monoclonal IgG (Sigma-Aldrich, St. Louis, MO, USA), anti-CLY polyclonal IgG (Cell Signaling Technology Inc., USA), anti-TNF*α* rabbit polyclonal IgG, anti-NF-*κ*B mouse monoclonal IgG, or anti-IL-10 goat polyclonal IgG (Santa Cruz Biotechnology, USA) diluted 1 : 500 in TTBS containing 3% dry albumin. GAPDH was included as a protein loading marker. Enhanced chemiluminescence was performed (SuperSignal West Pico, Pierce) after incubation with the appropriate horseradish peroxidase-conjugated secondary antibody.

### 2.7. Immunoblot Analysis of SREBP-1c and PPAR*γ* in Nuclear Extracts

Hepatic nuclear extracts were obtained as described by Siegrist-Kaiser et al. [36]. The protein concentrations of the nuclear extracts were determined by the Bradford assay [34]. Nuclear extracts (20 *μ*g) were separated by SDS-PAGE in 10% gels according to Milanski et al. [37]. The membranes were blocked with 5% skim milk in Tween-Tris-buffered saline (TTBS) (10 mmol/L Tris, 150 mmol/L NaCl, 0.5% Tween 20) overnight at 4°C. nSREBP-1c and PPAR*γ* were detected in the membranes after a 2 h incubation at room temperature with anti-SREBP-1c rabbit polyclonal IgG and anti-PPAR*γ* mouse polyclonal IgG (Santa Cruz Biotechnology; diluted 1 : 500 in TTBS containing 3% dry skimmed milk). Histone was used as a marker of the subcellular fraction and a protein loading marker. Enhanced chemiluminescence was performed (Super Signal West Pico, Pierce) after incubation with the appropriate horseradish peroxidase-conjugated secondary antibody.

### 2.8. RNA Preparation and Real-Time RT-PCR

Total RNA was isolated from frozen liver samples by TRIzol reagent (Invitrogen, USA), according to the supplier's instructions. Three micrograms of total RNA were transcribed into cDNA with High Capacity reverse transcriptase (Applied Biosystems). Primers specific for rat TNF*α* (Rn00563005_m1), IL-6 (Rn99999011_m1), IL-10 (Rn00563409_m1), NF-*κ*B (Rn01399583_m1), and glyceraldehyde-3-phosphate dehydrogenase (GAPDH) (Rn01775763_g1) were obtained from Applied Biosystems. GAPDH was used as the endogenous control. PCR was carried out in duplicate on a Step One system using TaqMan Gene Expression Master Mix (Applied Biosystems). The cDNA was amplified under the following conditions: 95°C for 10 min for denaturation and then 40 cycles of 95°C for 15 s, 60°C for 20 s, and 72°C for 15 s, followed by extension at 72°C for 10 min. Real-time data were analyzed using the Step One System (Applied Biosystems).

### 2.9. Statistical Analysis

The results are expressed as the means with their respective standard deviations for the number of rats indicated in parentheses. Bartlett's test for the homogeneity of variances was initially used to determine whether the data complied with the assumptions necessary for a parametric ANOVA. When necessary, the data were log transformed to correct for variance in heterogeneity or nonnormality [38]. A two-way ANOVA (i.e., effects of nutritional status in early life and diet) was used to compare the data from the CC, CS, LC, and LS groups. A one-way ANOVA was used to assess whether the diets were effective at improving the nutritional status of the LC, LS, and LL groups. When necessary, these analyses were complemented by the least significant difference test to determine the significance of the individual differences. *P* < 0.05 indicated statistical significance. All statistical comparisons were conducted using the Statistica software package (Stat-Soft).

## 3. Results

At the beginning of the recovery phase, LC, LS, and LL rats had similar body weights, and in all cases, these were significantly lower than those of the CC and CS rats. Body weight at the end of the experimental period was significantly lower in the LC and LS groups than in the CC and CS groups (*P* < 0.001). Rats maintained on a soybean flour diet after weaning (LS and CS groups) had a lower final body weight than those fed a casein diet (LC and CC groups) (*P* < 0.001). Although LS rats reached a higher final body weight than LL rats (*P* < 0.001), their weights were still significantly lower than those of LC rats (*P* < 0.001). Total serum protein concentrations did not differ among the CC, CS, LC, and LS groups. The LS and LC rats had higher total serum protein levels than rats from the LL group (*P* < 0.05). Serum triacylglycerol levels were higher in the LC group than in the LS, CS, and CC groups. In the LS group, serum triacylglycerol concentrations were lower relative to those of LC rats but did not differ from those exhibited by the LL rats. Serum insulin concentrations were higher in rats fed a soybean diet (CS and LS groups) than in rats fed a casein diet (CC and LC groups) (*P* < 0.0001). Basal serum glucose levels were not significantly different among the groups. The HOMA-IR index was higher in rats fed a soybean diet than in those reared on casein (*P* < 0.0001). Similar glycemia levels, but higher insulinemia and HOMA-IR indices, were recorded in LS rats relative to the LL and LC groups. There was no difference in HOMA-IR index or serum insulin concentrations between the LL and LC groups. Serum levels of alanine aminotransferase (ALT) were higher in the LS and LC groups than in the CS and CC groups (*P* < 0.01) and in rats maintained on a soybean diet (LS and CS rats) relative to those fed a casein diet (LC and CC rats) (*P* < 0.05). There was no difference in ALT levels between the LL, LS, and LC groups. Serum aspartate aminotransferase (AST) levels did not differ among the groups. Levels of serum gamma-glutamyl-transpeptidase (*γ*GT) in the LS group were significantly higher than those of the CS group but similar to those of the CC and LC groups. The LS rats had significantly higher *γ*GT levels than the LL rats, but they were similar to those of the LC group. Alkaline phosphatase (ALP) levels were significantly higher in the LS and LC groups than in the CS and CC groups (*P* < 0.01). The LS and LC groups showed ALP concentrations that were similar to each other and significantly lower than those observed in the LL group (Table 1).

**Figure 1:**
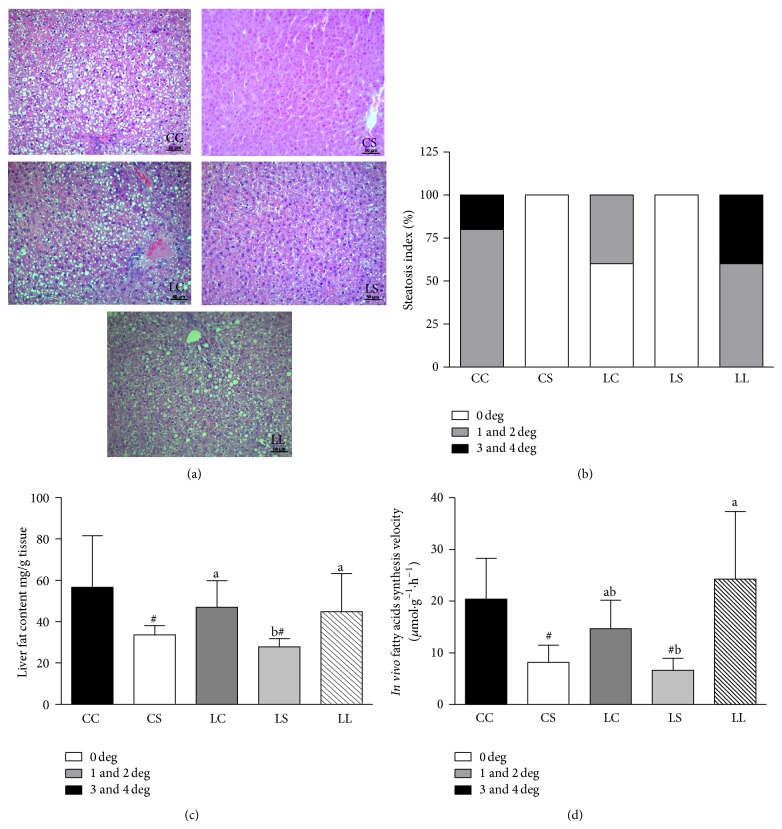
Representative photomicrographs of hematoxylin-eosin stained liver sections (a), steatosis index (b), liver fat content (c), and hepatic* in vivo* fatty acids synthesis rate (d) in adult rats maintained on control (CC and LC), soybean flour (CS and LS) or low-protein (LL) diets after weaning until 90 days of age. The values are expressed as the mean ± SD (*n* = 4–7 rats). ^#^Mean values were significantly different from the casein diet (two-way ANOVA). Means with different superscript minuscule letters are significantly different by one-way ANOVA followed by a least significant difference (LSD) test (*P* < 0.05).

Hepatocellular lipid accumulation (steatosis) was assessed in liver sections using the conventional hematoxylin-eosin staining technique (Figure 1(a)). Liver histology revealed that rats fed a soybean diet did not show an abnormal accumulation of fat in their livers because 100% of liver specimens from the LS and CS groups were graded as 0. In the LC group, 60% of liver specimens were graded as 0 and 40% as 1 or 2, whereas in the CC group, 80% were graded 1 or 2 and 20% as 3 or 4. In the LL group, 60% were graded 1 or 2 and 40% as 3 or 4 (Figure 1(b)). The hepatic fat content (Figure 1(c)) was reduced in rats fed a soybean diet (LS and CS groups) compared with rats fed a casein diet (LC and CC groups) (*P* < 0.001). The LS group had lower hepatic fat content than the LC and LL groups. Moreover, rats fed the soybean flour diet (CS and LS groups) exhibited lower fatty acid synthesis rates compared with those fed the casein diet (CC and LC rats) (*P* < 0.001). The fatty acid synthesis rate in the LS group was lower than that of the LL group and similar to that observed in the LC group (Figure 1(d)).

**Figure 2:**
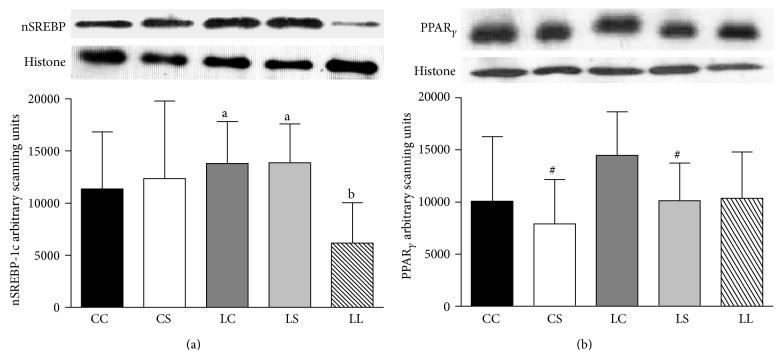
Immunoblot analysis of SREBP-1c (a) and PPAR*γ* (b) in nuclear extracts from livers of rats fed with casein (CC and LC), soybean-flour (CS and LS), or low-protein (LL) diets after weaning. The values are expressed as the mean ± SD (*n* = 4–7 rats). ^#^Mean values were significantly different from the casein diet (two-way ANOVA). Means with different superscript minuscule letters are significantly different by one-way ANOVA followed by a least significant difference (LSD) test (*P* < 0.05).

nSREBP-1c protein levels were similar across the CS, CC, LS, and LC groups. However, the LS and LC groups showed significantly higher nSREBP-1c than the LL group (Figure 2(a)). Rats fed the soybean diet (CS and LS groups) had lower nPPAR*γ* levels than those fed the casein diet (CC and LC groups) (*P* < 0.05). There was no difference in nPPAR*γ* levels among the LL, LS, and LC groups (Figure 2(b)).

**Figure 3:**
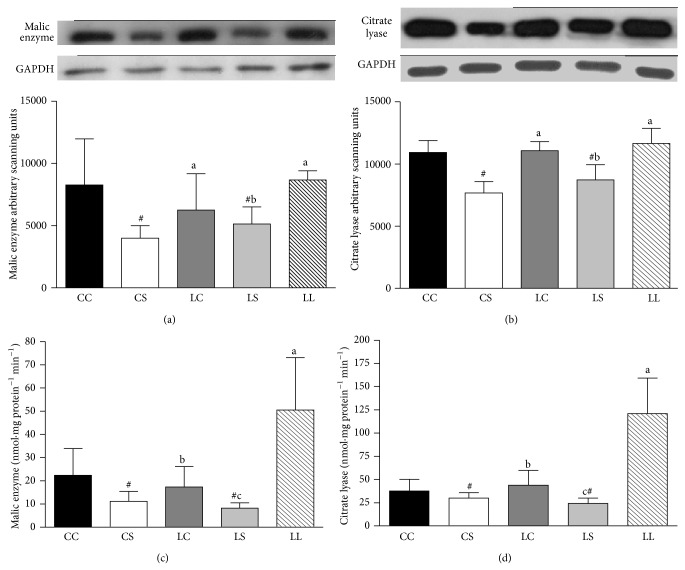
Immunoblot analysis of malic enzyme (a) and citrate lyase (b), activity of malic enzyme (c), and citrate lyase (d) in nuclear extracts from livers of rats fed with casein (CC and LC), soybean flour (CS and LS), or low-protein (LL) diets after weaning. The values are expressed as the mean ± SD (*n* = 4–7 rats). ^#^Mean values were significantly different from the casein diet (two-way ANOVA). Means with different superscript minuscule letters are significantly different by one-way ANOVA followed by a least significant difference (LSD) test (*P* < 0.05).

Liver ME and CLY contents were significantly lower in rats fed the soybean diet (CS and LS groups) relative to those fed the casein diet (CC and CS groups) (*P* < 0.05 and *P* < 0.01, resp.). Livers from the LS group also exhibited lower ME and CLY contents than livers from the LL and LC groups (Figures 3(a) and 3(b)). The CS and LS groups showed lower ME and CLY activities compared with the CC and LC groups (*P* < 0.05 and *P* < 0.01, resp.). Moreover, ME and CLY activities were lower in the LS rats than in the LC rats, and these activities were significantly lower in both groups relative to the LL rats (Figures 3(c) and 3(d)).

**Figure 4:**
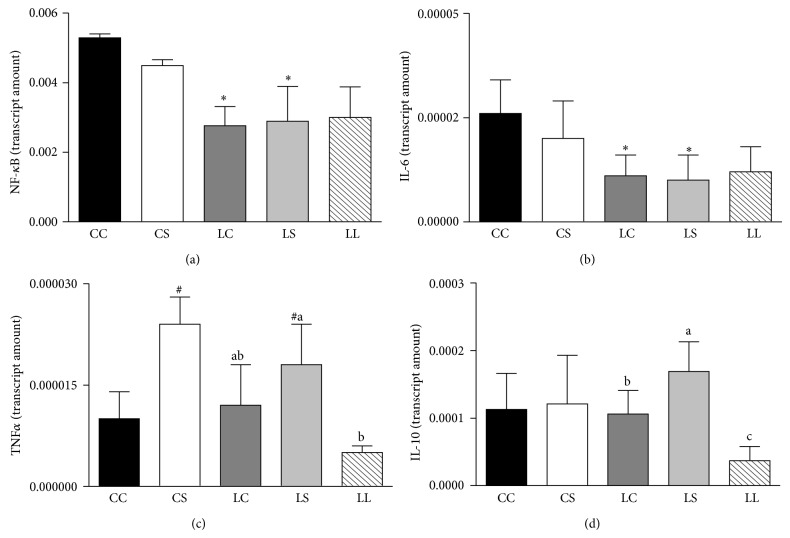
NF-*κ*B (a), IL6 (b), TNF*α* (c), and IL10 (d) mRNA analyzed by real-time polymerase chain reaction in livers of adult rats maintained on casein control (CC and LC), soybean flour (CS and LS), or low-protein (LL) diets after weaning. The values are expressed as the mean ± SD (*n* = 4–7 rats). ^#^Mean values were significantly different from the casein diet (two-way ANOVA). ^∗^Mean values were significantly different from rats born to mothers fed with 17% protein during pregnancy and lactation (two-way ANOVA). Means with different superscript minuscule letters are significantly different by one-way ANOVA followed by a least significant difference (LSD) test (*P* < 0.05).

mRNA levels of NF-*κ*B (Figure 4(a)) and IL-6 (Figure 4(b)) were significantly reduced in the LC and LS groups relative to the CC and CS groups (*P* < 0.0001; *P* < 0.005). No difference was observed in the NF-*κ*B and IL-6 mRNA expression among the LC, LS, and LL groups. TNF*α* mRNA levels (Figure 4(c)) were significantly higher in rats fed the soybean diet (LS and CS groups) than in those fed casein (LC and CC groups) (*P* < 0.01). The TNF*α* mRNA levels of the LS group were similar to those of the LC group and higher relative to the LL group (*P* < 0.05). IL-10 mRNA expression (Figure 4(d)) was not significantly different among the CC, CS, LC, and LS groups. However, the LS group exhibited significantly higher IL-10 mRNA expression than that observed in the LL and LC groups (*P* < 0.01).

**Figure 5:**
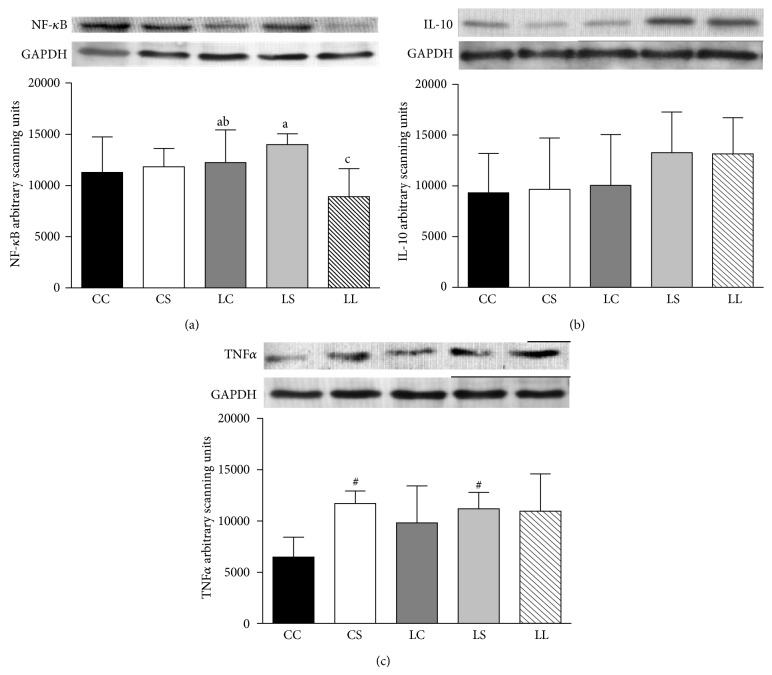
Immunoblot analysis of NF-*κ*B (a), IL10 (b), and TNF*α* (c) in livers of adult rats maintained on casein control (CC and LC), soybean flour (CS and LS), or low-protein (LL) diets after weaning. The values are expressed as the mean ± SD (*n* = 4–7 rats). ^#^Mean values were significantly different from the casein diet (two-way ANOVA). Means with different superscript minuscule letters are significantly different by one-way ANOVA followed by a least significant difference (LSD) test (*P* < 0.05).

NF-*κ*B (Figure 5(a)) and IL-10 (Figure 5(b)) protein levels did not differ among the CC, CS, LC, and LS groups. However, LS and LC rats exhibited higher NF-*κ*B protein levels than LL rats. No difference in IL-10 protein expression was observed among these groups. TNF*α* protein levels were higher in the CS and LS groups than in the CC and LC groups (*P* < 0.05), but no difference in TNF*α* levels was observed among the LL, LS, and LC groups (Figure 5(c)).

## 4. Discussion

In this study, rats recovered with the soybean diet showed low body weight and normalization of serum triacylglycerol concentrations, corroborating the well-known favorable effect of soy protein containing various levels of isoflavones on somatic parameters [39, 40] and serum triacylglycerol levels [41, 42]. However, contrary to the observations that soy protein reduces serum insulin levels [42, 43], we verify here, as in previous studies [23, 24, 26], that the consumption of a soybean flour diet increased serum insulin concentrations. This effect may be attributable to genistein, which increases insulin secretion due to its ability to activate the cAMP-PKA pathway [26].

Interestingly, our animals fed with soybean protein showed low liver fat content (assessed by gravimetric and histologic assays) and liver insulin resistance as determined by the HOMA-IR index and confirmed by high serum ALT concentrations (a hepatic enzyme whose appearance reflects hepatic insulin resistance and NAFLD) [44]. It is also noteworthy that abundant lipid droplets (observed histologically) as well as elevated liver fat contents were observed in the rats from the CC group. Hepatic steatosis has also been reported by others [45], who attributed this effect to the elevated amounts of carbohydrates and calories provided by the AIN-93 diet. However, the CC rats exhibited HOMA-IR index values and serum ALT concentrations not compatible with metabolic alterations or hepatocellular injury. Moreover, although liver fat contents appeared to be comparable between the CC and LL groups, twice as many liver specimens from the LL group were graded as 3 or 4.

Liver insulin resistance results in the overexpression of SREBP-1c [46], whereas soy protein [42] and genistein [47] suppress the SREBP-1c levels. Thus, in this and in a previous study [37], the interplay between liver insulin resistance, which induces SREBP-1c overexpression, and other factors (e.g., genistein and soy protein) that suppress its expression resulted in no change in nSREBP-1c contents or SREBP-1c mRNA expression. An adequate explanation for the paradoxical association of liver insulin resistance with unmodified nSREBP-1c concentrations is the mild hyperinsulinemia and the euglycemia observed in our animals fed with soybean, as noted here and previously [24]. SREBP-1c expression has been shown to be stimulated by glucose and insulin and repressed by glucagon [9, 10, 48]. Interestingly, the repression of SREBP-1c expression by soy protein has been attributed to decreased insulin : glucagon ratios [21]. In our animals, we previously observed reduced insulin : glucagon ratios in LC and LL rats compared with LS rats [24]. In that prior study [24], we observed that both the LC and LL groups exhibited lower serum insulin concentrations, lower liver insulin resistance, and basal glucose concentrations that were similar to those of LS rats. Surprisingly, only LL rats exhibited low nSREBP-1c levels, but LL and LC rats had similar liver fat storage levels, and both had higher liver fat content than LS rats. In contrast, we observed that the soybean diet resulted in reduced expression of PPAR*γ*, which targets the ME and CLY genes [49, 50]. In agreement with this finding and with the observation that soy protein reduces the expression of ME [42], we verified low contents of both ME and CLY in liver samples from rats fed the soybean diet. Interestingly, studies have shown that the genetic deletion of PPAR*γ* in the livers of lipodystrophic transgenic mice markedly attenuates the development of NAFLD, independent of the presence of hyperinsulinemia or hyperglycemia [50, 51].

Liver fat storage is also regulated by the integrated activities of cellular enzymes that catalyze lipid synthesis. Insulin acts as a key regulator of triglyceride biosynthesis in the liver by modulating enzymatic activities involved in the synthesis of fatty acids. In this study, both CLY and ME activities were reduced, and consequently, a decrease in* de novo* lipogenesis was observed in rats fed with soybean. It is well known that soy protein reduces ME activity in the liver [42]; however, in contrast to our observations, this suppressive effect was accompanied by low serum insulin concentrations and a reduced insulin : glucagon ratio [42]. In contrast to the animals fed soybean, in rats maintained on a low-protein diet, CLY and ME activity levels were elevated, resulting in an increase in the rate of* de novo* fatty acid synthesis, despite low levels of serum insulin. Two factors may have contributed to the increased activities of these enzymes: an increased sensitivity to insulin and the high carbohydrate levels of the low-protein diet. Some lines of evidence suggest that CLY and ME activity is stimulated by insulin and high carbohydrate intake [52, 53]. Thus, the high CLY and ME activity levels appeared to determine the elevated rate of* de novo* fatty acid synthesis in the livers of rats from the LL group.

Curiously, the animals that were fed a soybean diet did not exhibit lipid accumulation in hepatocytes but did have high serum ALT concentrations, a marker of liver injury [54]. A potential induction factor for the hepatocellular injury and fibrosis observed in NAFLD is oxidative stress [1]. It has been shown that chronic genistein supplementation at more than 500 mg/kg/day adversely affects liver structure and function [55]. Moreover, genistein increases the level and activity of PPAR*γ* [22, 56], which, in the liver, plays a pivotal role in fatty acid catabolism by upregulating the expression of numerous genes involved in mitochondrial and peroxisomal fatty acid oxidation [57]. Consequently, activation of PPAR*γ* can prevent and decrease hepatic fat storage [58], but the oxidation of fatty acids remains an important source of reactive oxygen species (ROS) in fatty livers [59]. This hypothesis is weakened by the fact that the amount of genistein contained in our diet was determined to be lower than 500 mg/kg body weight/day [23], yet our soybean-fed animals exhibited reduced PPAR*γ* mRNA expression and unchanged PPAR*γ* protein contents in the liver. However, these animals also exhibited reduced expression of liver acetyl-coenzyme A carboxylase beta (ACC*β*) [37], an enzyme localized in the mitochondrial membrane, where it is believed to regulate local malonyl-coenzyme A levels, carnitine palmitoyltransferase I (CPT-1) activity, and fat oxidation. The lack of ACC*β* is associated with continuous fatty acid oxidation and reduced fat storage [60]. We also previously observed a reduction in nonprotein thiol levels in the livers of soybean-treated animals, indicating the consumption of nonprotein thiols by enhanced free radical generation (unpublished data).

A consequence of increased ROS includes the release of proinflammatory cytokines [2]. Hence, elevated ALT may reflect inflammation, which impairs insulin signaling in the liver. However, ALT is also a gluconeogenic enzyme, and increased ALT levels could therefore indicate impaired insulin signaling instead of liver injury. In our rats that were fed the soybean diet, the increased ALT, combined with high TNF*α* mRNA and protein levels, is consistent with inflammation. However, the unchanged NF-*κ*B expression in livers from these soybean-fed rats is indicative of the absence of fibrosis because NF-*κ*B is considered a profibrotic marker [61]. The rats that were rescued from malnutrition in early life showed reduced transcription of NF-*κ*B and IL-6 and increased transcription of IL-10 (an anti-inflammatory cytokine). However, neither NF-*κ*B nor IL-10 protein levels were altered under these conditions. Given the elevated TNF*α* content, it is reasonable to suggest that the soybean diet increased NF-*κ*B activity. Interestingly, in the livers of rats maintained on the soybean diet, the expression of TNF*α* was correlated with the HOMA-IR index, confirming the role of this cytokine in the development of hepatic insulin resistance [62].

In conclusion, the present study showed that a soybean diet prevented steatosis at least in part through reduced lipogenesis but resulted in TNF*α*-mediated inflammation.

## Figures and Tables

**Table 1:** Somatic, biochemical, and hormonal parameters from adult rats maintained on control (CC and LC groups) soybean flour (CS and LS groups), or low protein (LL group) diet after weaning until 90 days age.

Variables	Groups				
	CC	CS	LC	LS	LL
Body weight (g)					
Initial	78 ± 13 (10)	71 ± 11 (10)	24 ± 6^*^ (12)	23 ± 3^*^ (12)	28 ± 3 (13)
Final	434 ± 53 (10)	364 ± 20^#^ (11)	333 ± 23^∗a^ (12)	263 ± 13^#∗b^ (12)	154 ± 34^c^ (13)
Serum total protein (g/L)	56 ± 2 (6)	55 ± 3 (7)	58 ± 3^a^ (11)	56 ± 2^a^ (7)	50 ± 3^b^ (6)
Serum triglycerides (mmol/L)	0.6 ± 0.3^B^ (5)	0.6 ± 0.1^B^ (5)	1.2 ± 0.5^Aa^ (7)	0.6 ± 0.1^Bb^ (7)	1.0 ± 0.4^ab^ (6)
Serum glucose (mmol/L)	3.9 ± 1.2 (9)	4.7 ± 1.1 (7)	4.2 ± 1.1 (10)	4.5 ± 1.0 (7)	4.2 ± 1.8 (8)
Serum insulin (*μ*U/mL)	12 ± 4 (9)	33 ± 8^#^ (7)	11 ± 3^b^ (10)	26 ± 10^#a^ (7)	13 ± 3^b^ (8)
HOMA-IR	2.1 ± 0.7 (9)	6.6 ± 1.5^#^ (7)	2.2 ± 0.9^b^ (10)	5.3 ± 2.6^#a^ (7)	2.5 ± 1.4^b^ (8)
ALT (U/L)	28 ± 6 (5)	43 ± 8^#^ (5)	45 ± 13^*^ (7)	51 ± 12^#∗^ (7)	54 ± 6 (6)
AST (U/L)	104 ± 25 (5)	163 ± 55^#^ (5)	177 ± 64^*^ (7)	177 ± 54^#∗^ (7)	208 ± 39 (6)
*γ*GT (U/L)	9 ± 2^AB^ (5)	6 ± 4^B^ (5)	8 ± 3^ABab^ (7)	10 ± 2^Aa^ (7)	6 ± 3^b^ (6)
ALP (U/L)	144 ± 21 (5)	141 ± 16 (5)	178 ± 33^∗b^ (7)	199 ± 27^∗b^ (7)	492 ± 66^a^ (6)

The values are the mean ± SD of the number of rats indicated in parentheses. ^#^Mean values were significantly different from the control rats (*P* < 0.05; two-way ANOVA). ^*^Mean values were significantly different from the rats fed a casein diet (*P* < 0.05, two-way ANOVA). Means with different superscript capital letters are significantly different by two-way ANOVA and with superscript minuscule letters are significantly different by one-way ANOVA followed by LSD test (*P* < 0.05).
